# Uncertainty-Aware structured decoding for Chinese Braille document transcription

**DOI:** 10.1371/journal.pone.0359339

**Published:** 2026-09-25

**Authors:** Yu Sun, Wenhao Chen, Yihang Qin, Wenhui Zhao, Haoran Sun

**Affiliations:** 1 School of Special Education, Changchun University, Changchun, China; 2 School of Computer Science and Technology, Changchun University, Changchun, China; University of Hyderabad, INDIA

## Abstract

Automatic transcription of braille documents remains challenging in document digitization settings, particularly under real imaging degradations such as uneven embossing, illumination variation, paper texture, skew, blur, and ghosting, which make braille dot presence uncertain and often cause error propagation from dot detection to cell encoding and final text transcription. To address this problem, we propose an uncertainty-aware structured decoding framework for Chinese braille document transcription that formulates the task as document analysis with an explicit intermediate representation rather than as a purely end-to-end black-box mapping. The method first performs robust grid fitting and structured dot analysis within braille candidate regions, and converts dot responses into calibrated probabilities. Topological consistency constraints are then imposed to recover valid six-dot cell patterns while producing dot-level and cell-level confidence estimates for risk localization. To resolve residual ambiguity efficiently, only low-confidence cells are expanded into small local candidate sets, which are rescored using contextual compatibility and local structural cost. Experiments on authentic scanned and photographed braille documents using document-level five-fold cross-validation achieved a Dot-F1 of 0.992 ± 0.002, a character error rate of 0.018 ± 0.003, and a BLEU-4 score of 74.1 ± 1.2. The results demonstrate that the proposed framework improves structural recovery stability, transcription accuracy, and sentence-level consistency, while maintaining strong robustness across document conditions and limiting additional inference overhead.

## 1. Introduction

Braille documents are still widely used in education, public services, and archival records, and reliable transcription is important for document digitization and accessibility applications [1,2]. Unlike conventional OCR, however, braille transcription is based on a fixed six-dot cell arranged in a 2 × 3 grid. This structure makes the task highly sensitive to local visual errors, because a single missing or spurious dot may change the cell code and then affect the final text output [2,3].

In real scanned or photographed braille documents, dot detection is often unstable. Uneven embossing may weaken part of the dot response, while illumination variation, paper texture, bleed-through, skew, blur, and ghosting can introduce false responses or disturb the regular lattice structure [3–8]. These factors make it difficult to decide whether a dot is truly present. Existing end-to-end braille transcription methods usually map the image directly to text [9–15]. Although such methods can work well on relatively clean data, their performance may vary across document conditions, and errors are often difficult to trace back to either the visual parsing stage or the text generation stage.

This problem suggests that braille transcription should not be treated only as a direct image-to-text task. Recent studies have shown that uncertainty-aware probabilistic inference can improve robustness and reliability in document analysis under heterogeneous scanned-document conditions [16]. Context-aware OCR and reconstruction have also been used to reduce cascading errors in structured document digitization [17]. These ideas are relevant to braille documents because braille pages have strong structural regularity, including fixed cell topology, constrained spatial arrangement, and stable reading order.

Based on this observation, Chinese braille transcription is formulated here as a structured decoding problem in which visual structural recovery is separated from sequence-level text disambiguation. The proposed framework first performs grid fitting and structured dot analysis in candidate braille regions, and then converts dot responses into calibrated probabilities. Topological consistency constraints are used to recover valid six-dot cell patterns and to produce dot-level and cell-level confidence estimates. Low-confidence positions are further handled by local candidate expansion and contextual rescoring. In this way, uncertainty is handled before final text generation rather than after it. The study makes three main contributions: it introduces an explicit intermediate representation for Chinese braille transcription, develops a confidence-guided local disambiguation strategy to limit error propagation with modest computational cost, and provides a document-level evaluation protocol including ablation, robustness, sensitivity, and efficiency analysis.

Furthermore, understanding braille structural recovery computationally parallels human tactile-language processing and braille reading networks. Bridging visual decoding with structural consistency not only aids document digitization but also supports broader accessibility goals, promoting tactile literacy and bilingualism among the visually impaired. By quantifying sensory-level uncertainty before semantic interpretation, our framework loosely mirrors the sensory perception mechanisms observed in blind readers.

## 2. Related work

Existing studies on braille recognition can be roughly grouped into three lines: rule-based visual decoding, learning-based dot or character recognition, and end-to-end transcription. The main differences among them lie in how much structural prior they use, how they handle imaging degradation, and whether errors can be localized and interpreted.

Rule-based methods make direct use of the geometric regularity of braille. Typical pipelines decompose the task into dot candidate extraction, row and column grouping, grid fitting, and six-dot code determination. Their main advantage is clarity. When recognition fails, the source of the error can often be traced to a specific stage, such as poor grid estimation or unstable thresholding. The weakness is that these methods depend heavily on hand-crafted rules and are usually sensitive to document condition. Variations in embossing depth, paper texture, ghosting, and other imaging artifacts can easily shift response statistics and make parameters less stable across documents [3,4,8–20]

Learning-based methods improve local feature extraction at the dot or character level and usually show better tolerance to complex imaging conditions than purely rule-based pipelines [5–8,21]. This is especially useful when dot appearance is affected by blur, uneven lighting, or background interference. Still, local prediction alone does not solve the whole problem. If dot or character decisions are made independently, without explicit constraints from the six-dot structure or row-column layout, small visual errors can still propagate to cell decoding and then to the final text output.

End-to-end methods shorten the pipeline by mapping braille images directly to text. This reduces the amount of manual engineering and allows contextual information to be used during decoding. In practice, however, braille is a highly discrete visual system with strong geometric constraints, so end-to-end models must handle structural alignment and semantic generation at the same time. This can make performance less stable when document conditions change, and it also makes error analysis harder because structural errors and language-level errors are mixed in the same model [7,9–15]

Recent work outside braille-specific pipelines is also relevant here. Uncertainty-aware probabilistic inference has been shown to improve robustness and reliability in document analysis under heterogeneous scanned-document conditions [16]. Context-aware OCR and reconstruction have also been used to reduce cascading recognition errors in structured document digitization [17]. These two directions are closely related to braille transcription, where local visual uncertainty and downstream decoding errors are tightly coupled. What remains insufficient in the current braille literature is a framework that keeps structural recovery explicit, quantifies uncertainty at the intermediate stage, and uses that uncertainty to guide later disambiguation rather than leaving the full burden to a black-box model.

## 3. Prerequisite knowledge and notation

### 3.1. Six-Dot Braille and intermediate representation

Six-dot Braille is a highly structured discrete encoding system, with the 2 × 3 dot matrix unit as its smallest semantic carrier. Each braille cell is represented as a six-digit vector within a finite discrete state space, denoted as $b\in {\left\{\text{0},\text{1}\right\}}^{\text{6}}$, where $b=(d_{\text{1}},d_{\text{2}},d_{\text{3}},d_{\text{4}},d_{\text{5}},d_{\text{6}})$ and $d_{i}\in \{\text{0},\text{1}\}$ represents the presence state of i-th dot. Consequently, the cell-level structural space is constrained within a finite discrete set, with at most ${\text{2}}^{\text{6}}$ possible patterns [2]. Braille cells do not exist in isolation but form text sequences through row-column structures. By unfolding cells within a braille region of interest (ROI) in reading order, a six-digit sequence $B=(b_{\text{1}},\ldots ,b_{L})$ is obtained, where L represents the total number of cells within the region.

*B* serves as the core intermediate representation linking braille images to Chinese text, decomposing the braille transcription conditional inference problem, from ROI image R to Chinese text Y, into two decoupled subproblems: structural reconstruction and semantic generation. The value of this decomposition lies in explicitly exposing and quantifying visual-side uncertainties, such as dot presence and geometric alignment, during the structural reconstruction stage, while uncertainties on the semantic side, such as ambiguous mappings and contextual selection, are handled during text generation. In this way, error attribution problems caused by mixing different types of uncertainty in a black-box model can be reduced. This “represent first, map later” strategy is also common in Chinese Braille translation systems and end-to-end image-to-text methods [9–15].

Under real imaging conditions, the presence of braille dots is not strictly deterministic. Therefore, beyond binary six-code encoding, a confidence representation is introduced by defining B as a structured state variable that also carries confidence information. This confidence representation serves two purposes: it guides the selection of structural repair strategies and triggers local disambiguation as well as computational budget allocation at the sequence level, thereby forming a closed loop among structural recovery, semantic generation, and error localization. Research on uncertainty quantification provides an overarching framework for this idea [22,23]; calibration methods support consistency between probabilistic outputs and true accuracy [24,25]; distribution-free confidence control offers a basis for risk-constrained inference [26]; and training methods for uncertainty-aware classifiers provide more implementation-oriented references [27].

### 3.2. Decomposition mapping and training objectives

Given a document image I, front-end localization yields a set of braille candidate regions $R=R_{m}$, where $R_{m}$ represents the m-th ROI corresponding to a segment of local braille content. A structure-first, semantics-later strategy is adopted here, decomposing braille transcription into the following two-stage mapping:


$$\begin{array}{c} \begin{array}{c} R_{m}\overset{f_{\theta }}{⟶}B_{m}\overset{g_{\psi }}{⟶}Y_{m} \end{array} \end{array}$$ (1)

Where $f_{\theta }$ is the structured dot matrix parser, which outputs the braille six-dot sequence $B_{m}$ together with its confidence information, and $g_{\psi }$ is the sequence generation and disambiguation module, which maps the six-dot sequence to a Chinese text sequence $Y_{m}$. This decomposition makes structural-side errors, such as missed dots or misaligned cells, directly measurable and easier to localize. Such errors can be corrected through structural consistency constraints before semantic generation. On the semantic side, candidate expansion is required only at a small number of low-confidence positions, which helps prevent dot-level errors from propagating across layers to the text level.

Model training adopts joint optimization of point-level and sequence-level supervision, so that structural reconstruction error and end-to-end transcription error are minimized together. The joint objective function is:


$$\begin{array}{c} \begin{array}{c} {min}_{\theta ,\psi }\;L(\theta ,\psi )=\lambda_{dot}L_{dot}(f_{\theta })+\lambda_{seq}L_{seq}(g_{\psi }\circ f_{\theta }) \end{array} \end{array}$$ (2)

$\lambda_{dot}$ and $\lambda_{seq}$ serve as weighting coefficients to balance the loss contribution between structural learning and text generation; $L_{dot}$ denotes the point-level loss, which measures the accuracy of dot structure reconstruction, and $L_{seq}$ denotes the sequence-level loss, which measures end-to-end text transcription accuracy.

### 3.3. Unified evaluation metric definition

To ensure reproducibility, comparability, and hierarchical diagnostic of experimental results, three evaluation metrics are used in this study, corresponding to structural recovery, character-level transcription accuracy, and sentence-level consistency. Together, these metrics provide a multi-level view of system performance from dot structure to final text output. Consistent metric definitions are particularly important in document-level transcription tasks, where changes at the structural level may propagate to later stages and affect end-to-end interpretation [28,29]. Sentence-level metrics should therefore be interpreted together with dot-level and character-level results rather than in isolation [30].

**(1) Dot-level: Dot-F1**

Each dot position in a braille cell is treated as a binary classification target. Let Ω denote the set of all dot positions, let $d_{l,i}$ denote the ground-truth label of the i-th dot in the l-th cell, and let ${\hat{d}}_{l,i}$ denote the predicted label. True positives (TP), false positives (FP), and false negatives (FN) are defined as follows:


$$\begin{array}{c} \begin{array}{c} \{TP=\left|\left\{(l,i)\in \Omega :d_{l,i}=\text{1}\wedge {\hat{d}}_{l,i}=\text{1}\right\}\right| \end{array} \end{array}$$ (3)

$$\begin{array}{c} \begin{array}{c} \{FP=\left|\left\{(l,i)\in \Omega :d_{l,i}=\text{0}\wedge {\hat{d}}_{l,i}=\text{1}\right\}\right| \end{array} \end{array}$$ (4)

$$\begin{array}{c} \begin{array}{c} \{FN=\left|\left\{(l,i)\in \Omega :d_{l,i}=\text{1}\wedge {\hat{d}}_{l,i}=\text{0}\right\}\right| \end{array} \end{array}$$ (5)

Based on these definitions, Precision and Recall are calculated as:


$$\begin{array}{c} \begin{array}{c} Precision=\frac{TP}{TP+FP},\;Recall=\frac{TP}{TP+FN} \end{array} \end{array}$$ (6)

Dot−F1 is the harmonic mean of Precision and Recall:


$$\begin{array}{c} \begin{array}{c} Dot-F\text{1}=\text{2}\cdot \frac{Precision\cdot Recall}{Precision+Recall} \end{array} \end{array}$$ (7)

Dot−F1 directly reflects the quality of dot-matrix structure recovery and provides a structural lower bound for subsequent text transcription accuracy.

**(2) Character Level: CER (Character Error Rate)**

Character-level differences between predicted text and reference text are measured by edit distance, The Character Error Rate (CER) is formally defined as:


$$\begin{array}{c} \begin{array}{c} CER=\frac{S+D+I}{N} \end{array} \end{array}$$ (8)

Where S, D and I denote the numbers of substitutions, deletions, and insertions, respectively, and N denotes the total number of reference characters (N=S+D+C, where C is the number of correctly recognized characters). CER directly reflects the practical usability of end-to-end braille transcription, with lower values indicating higher transcription accuracy.

**(3) Sentence Level: BLEU-4**

BLEU−4 is used to measure sentence-level matching and local contextual consistency through n-gram overlap:


$$\begin{array}{c} BLEU-\text{4}=BP\cdot exp\left(\frac{\text{1}}{\text{4}}\cdot \sum_{n=\text{1}}^{\text{4}}logp_{n}\right) \end{array}$$ (9)

Where $p_{n}$ is the n-gram precision and BP is the brevity penalty used to correct length differences between predicted and reference texts. BLEU−4 is sensitive to phrase-level consistency and is useful for evaluating local matching quality after sequence disambiguation [28]. In this study, BLEU−4 is not used alone, but is reported together with Dot−F1 and CER so that sentence-level performance can be interpreted together with structural and character-level results.

## 4. Method

**Fig 1:**
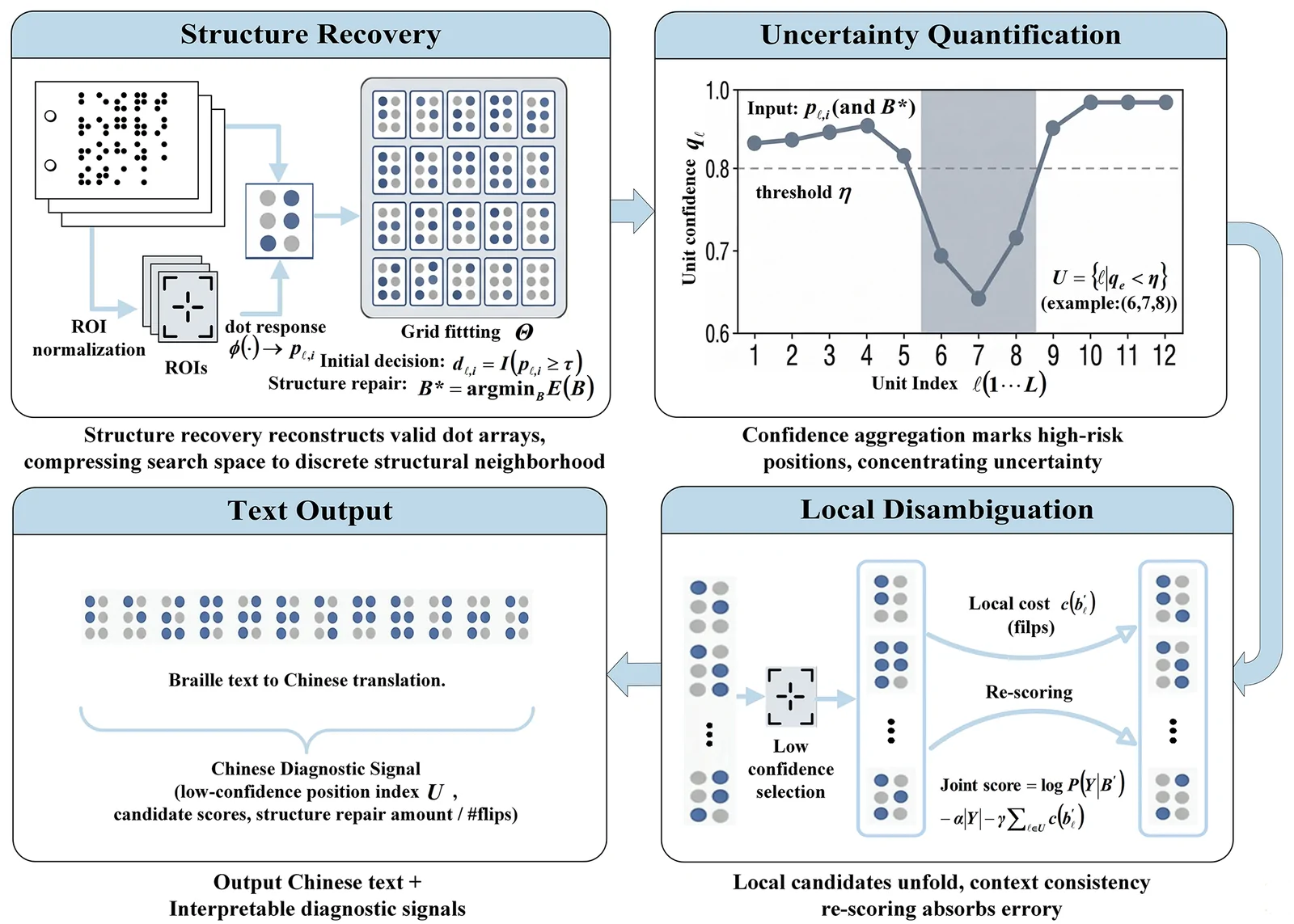
Overview of the proposed closed-loop framework for Chinese braille transcription.

The proposed framework treats braille transcription as a structured inference process rather than a direct pixel-to-text mapping. As shown in Fig 1, the full pipeline is organized into four connected parts: structural recovery, uncertainty estimation, local disambiguation, and text generation. The main idea is to first reduce the continuous visual problem to a constrained six-dot structural representation, and then resolve the remaining ambiguity only where the structural evidence is weak. In this way, most visual noise is handled before text generation, while additional computation is concentrated on a small number of difficult positions. The framework outputs not only the final Chinese text, but also intermediate signals such as confidence scores, low-confidence positions, and candidate-level scores, which are useful for error tracing and manual verification.

### 4.1. ROI normalization and point response function

The first step is to improve the separability between braille dots and background within each ROI. A lightweight normalization process is applied to suppress low-frequency illumination variation, reduce interference from paper texture and show-through, and preserve the local shape of raised dots. The purpose of this step is not to alter the geometric structure of the braille pattern, but to make subsequent dot responses more stable under different document conditions.

After normalization, a dot response map S(x,y) is computed over the ROI by a point response function φ(·). Higher response values indicate that the corresponding pixel location is more likely to be the center of a braille dot. This response function can be implemented either by a rule-based geometric operator or by a lightweight learned detector, but in both cases its output is interpreted in the same way: as local evidence for dot existence.

To support uncertainty-aware inference, the response values are converted into point-existence probabilities:


$$\begin{array}{c} \begin{array}{c} p=\sigma \left(\frac{S(x,y)-\beta }{T}\right) \end{array} \end{array}$$ (10)

Where σ(·) is the sigmoid function, β is a bias term used to compensate for response drift across document domains, and T is a temperature parameter controlling the sharpness of the probability distribution. Calibration is required so that the resulting probabilities remain consistent with actual prediction reliability, rather than becoming systematically overconfident or overly conservative.[24,25]

### 4.2. Grid parameterization and robust fitting

Braille cells follow a regular row-column arrangement. For structured analysis in the continuous image domain, a sampling coordinate system consistent with the six-dot layout is first defined. The grid parameters are written as $𝛩=(x_{\text{0}},y_{\text{0}},s_{x},s_{y},\theta )$, where$\left(x_{\text{0}},y_{\text{0}}\right)$ denotes the grid origin, $s_{x}$ and $s_{y}$ denote the horizontal and vertical spacing, and θ denotes the grid rotation angle.

Based on the grid parameter Θ and the standard relative coordinates $\left({\text{u}}_{\text{𝓁},i},{\text{v}}_{\text{𝓁},i}\right)$ of a 2 × 3 dot matrix, the theoretical coordinates of the i-th dot position in the 𝓁-th cell can be calculated:


$$\begin{array}{c} \begin{array}{c} {\text{x}}_{\text{𝓁},i}={\text{x}}_{\text{0}}+{\text{s}}_{\text{x}}\cdot {\text{u}}_{\text{𝓁},i}\cdot \text{cos}\theta -{\text{s}}_{\text{y}}\cdot {\text{v}}_{\text{𝓁},i}\cdot \text{sin}\theta \end{array} \end{array}$$ (11)

$$\begin{array}{c} \begin{array}{c} {\text{y}}_{\text{𝓁},i}={\text{y}}_{\text{0}}+{\text{s}}_{\text{x}}\cdot {\text{u}}_{\text{𝓁},i}\cdot \text{sin}\theta +{\text{s}}_{\text{y}}\cdot {\text{v}}_{\text{𝓁},i}\cdot \text{cos}\theta \end{array} \end{array}$$ (12)

The purpose of grid fitting is to align these theoretical dot locations with high-response regions in the point response map. In this way, the continuous image signal is converted into a sampling structure that is consistent with braille geometry. Since real document images often contain spurious peaks, bleed-through, and local noise, direct least-squares fitting is not sufficiently stable. A robust fitting objective is therefore used:


$$\begin{array}{c} \begin{array}{c} \text{mi}{\text{n}}_{\Theta }\;\Sigma_{\text{𝓁},i}\;\rho (-\text{S}({\text{x}}_{\text{𝓁},i},{\text{y}}_{\text{𝓁},i})) \end{array} \end{array}$$ (13)

Where ρ(·) denotes a robust loss function, such as the Huber loss or a smooth softplus-type penalty. This formulation reduces the influence of outliers while preserving the dominant geometric pattern of the braille lattice. In practice, optimization is carried out in a coarse-to-fine manner: a global search is first used to obtain a stable initialization, and local refinement is then applied to improve alignment. This strategy is more stable than direct local optimization and remains suitable for deployment when the number of iterations is kept finite.

### 4.3. Point sampling, initial classification, and calibration

After the grid parameters have been determined, point responses are sampled at the theoretical dot coordinates and converted into point-existence probabilities. In this way, the continuous response map is reduced to a set of discrete dot-level observations aligned with the braille lattice.


$$\begin{array}{c} \begin{array}{c} {\text{s}}_{\text{𝓁},\text{i}}=\text{S}({\text{x}}_{\text{𝓁},i},{\text{y}}_{\text{𝓁},i}) \end{array} \end{array}$$ (14)

$$\begin{array}{c} \begin{array}{c} {\text{p}}_{\text{𝓁},\text{i}}=\sigma \left(\frac{{\text{s}}_{\text{𝓁},i}-\beta }{\text{T}}\right) \end{array} \end{array}$$ (15)

Based on the resulting probabilities, an initial binary decision is made for each sampled point using a threshold τ:


$$\begin{array}{c} \begin{array}{c} {\hat{\text{d}}}_{\text{𝓁},i}=I({\text{p}}_{\text{𝓁},i}\geq \tau ) \end{array} \end{array}$$ (16)

Where I(·) denotes the indicator function, which equals 1 when the condition is satisfied and 0 otherwise. This step provides an initial point-level estimate of the six-dot structure before any structural repair is applied.

A fixed threshold is simple to use, but its behavior may vary across document domains because response statistics are affected by embossing depth, lighting, paper texture, and background interference. For this reason, threshold selection needs to account for domain variation. In practice, two choices are possible. One is to use an adaptive threshold derived from ROI-level response statistics. The other is to select a fixed threshold on the validation set when cross-domain stability is preferred. The latter is often easier to control in evaluation, while the former can be more flexible under changing image conditions.

### 4.4. Structural consistency constraint: Energy minimization formulation

Independent point detection is sensitive to noise and may therefore amplify local point-level errors. For this reason, the initial binary decisions are not used directly as the final six-dot sequence. Instead, structural repair is introduced through topological consistency constraints, and the optimal sequence is obtained by minimizing an energy function. The optimal braille sequence configuration $B^{*}$ is obtained by minimizing the global energy function over the valid structural hypothesis space:


$$\begin{array}{c} B^{*}=\underset{B\in B}{\text{arg}\text{min}}E(B) \end{array}$$ (17)

Where B denotes the valid hypothesis space of all possible six-dot sequence configurations. The energy function E(B) comprises a univariate term, a structural term, and a smoothing term, comprehensively considering the confidence of point predictions, structural constraints of the six-dot sequence, and local consistency of the sequence:


$$\begin{array}{c} \begin{array}{c} E(B)=\Sigma_{\text{𝓁},i}w_{\text{𝓁},i}\cdot I\left[{\text{b}}_{\text{𝓁},i}\text{≠}{\hat{\text{d}}}_{\text{𝓁},i}\right]+\lambda \cdot E_{struct}(B)+\mu \cdot E_{smooth}(B) \end{array} \end{array}$$ (18)

In this formulation, the penalty weight is defined strictly as a complementary function of the probability $w_{\text{𝓁},i}=\text{1}-{\text{p}}_{\text{𝓁},i}$ where ${\text{p}}_{\text{𝓁},i}$ is the calibrated existence probability of the i-th dot in the 𝓁-th cell, so that positions with lower confidence receive larger weights and are given higher priority during structural correction. $E_{struct}(B)$ is the structural term, which enforces braille-specific priors such as penalties on invalid six-dot combinations and other implausible local configurations. $E_{smooth}(B)$ is the smoothing term, which discourages abrupt changes between adjacent cells and helps preserve local sequence consistency. The coefficients λ and μ control the relative contributions of the structural and smoothing terms.

This formulation allows the initial point predictions to be revised under explicit structural constraints rather than treated as independent local decisions. As a result, the recovered six-dot sequence remains more consistent with braille topology and is less affected by isolated noisy responses or local sampling errors.

### 4.5. Uncertainty aggregation and risk localization

To support subsequent sequence-level disambiguation, point-level confidence is aggregated into cell-level confidence so that high-risk braille cells can be identified explicitly. A conservative aggregation rule is used here: the confidence of a cell is defined as the minimum point-existence probability among all points within that cell. This avoids masking a low-confidence point by averaging it with several high-confidence points:


$$\begin{array}{c} \begin{array}{c} {\text{q}}_{\text{𝓁}}=\text{mi}{\text{n}}_{\text{i}}{\text{ p}}_{\text{𝓁},\text{i}} \end{array} \end{array}$$ (19)

Other aggregation rules, such as the product or geometric mean, may also be used when a different balance between sensitivity and stability is needed.

Based on the cell-level confidence $q_{\text{𝓁}}$, a low-confidence set U is defined to select positions that require candidate expansion:


$$\begin{array}{c} \begin{array}{c} U=\{\text{𝓁}|q_{\text{𝓁}}<\eta \} \end{array} \end{array}$$ (20)

Where η is the disambiguation trigger threshold. This threshold controls how often local disambiguation is activated and therefore directly affects computational overhead.

To assess whether the uncertainty measure is useful in practice, two diagnostic quantities are introduced. Coverage Cover measures the proportion of text associated with the low-confidence set relative to the full text sequence, and Error Concentration Err@U measures the proportion of editing errors that fall within the low-confidence set. Their definitions are given by:


$$\begin{array}{c} \begin{array}{c} \text{Cover}=\frac{\left|\{\text{t}:{\text{y}}_{\text{t}}\text{ is mapped from }U\}\right|}{\left|\text{Y}\right|} \end{array} \end{array}$$ (21)

$$\begin{array}{c} \begin{array}{c} \text{Err}@\text{U}=\frac{\left(\#\text{ of edits occurring in region }U\right)}{\text{S}+\text{D}+\text{I}} \end{array} \end{array}$$ (22)

Higher values of these two quantities indicate that uncertainty estimation is more effective at locating positions that are likely to cause downstream transcription errors.

### 4.6. Candidate expansion and local cost

To avoid the combinatorial growth caused by full-sequence candidate expansion, candidate generation is restricted to cells in the low-confidence set U. For each such cell, only a small n-best candidate set is constructed, and only a limited number of dot flips is allowed so that the candidate space remains manageable.

For any candidate encoding ${\text{b}}_{\text{𝓁}}^{\prime }$, a local cost is defined relative to the initial decision in order to measure how far the candidate deviates from the original structural estimate:


$$\begin{array}{c} \begin{array}{c} \text{c}({\text{b}}_{\text{𝓁}}^{\prime })=\Sigma_{i}{\text{w}}_{\text{𝓁},i}\cdot I[{\text{b}}_{\text{𝓁},i}^{\prime }\text{≠}{\hat{\text{d}}}_{\text{𝓁},i}] \end{array} \end{array}$$ (23)

A smaller local cost means that the candidate remains closer to the initial judgment and is therefore more plausible unless stronger contextual evidence suggests otherwise. Candidates are sorted in ascending order of local cost, and the top n candidates are retained as the candidate set for that cell. This keeps candidate generation local and controlled, rather than allowing uncertainty at a few positions to expand into a global search problem.

Under this design, the total number of candidate combinations for the full six-dot sequence can be approximated by:


$$\begin{array}{c} \begin{array}{c} {\text{N}}_{\text{cand}}\approx \text{1}+\text{r}\cdot \text{L}\cdot (n-\text{1}) \end{array} \end{array}$$ (24)

Where r=|U|/L denotes the disambiguation trigger ratio. This expression makes the cost of candidate expansion directly dependent on the number of low-confidence cells rather than on the full sequence length. By adjusting the trigger threshold η and the candidate number n, the trade-off between transcription accuracy and computational overhead can be controlled in a simple and explicit way. This design is also consistent with the efficiency analysis in the experimental section, where sequence disambiguation contributes only a limited fraction of the total inference time.

### 4.7. Sequence-level disambiguation: Joint scoring of structure and semantics

After structural recovery, the remaining ambiguity is usually concentrated in a small number of low-confidence cells rather than being distributed across the whole sequence. For this reason, sequence-level disambiguation is applied only to the low-confidence set U, instead of expanding candidates for all braille cells. This keeps the search space manageable and avoids unnecessary computation on positions that are already structurally reliable.

For each selected cell, a small candidate set is constructed from alternative six-dot patterns that remain plausible under the local confidence distribution. These candidates are then mapped to text candidates and evaluated jointly in context. The rescoring step combines two sources of evidence: contextual compatibility, which favors candidates that better fit the surrounding sequence, and local structural cost, which penalizes candidates that deviate more strongly from the recovered dot pattern.

The joint score is defined as follows:


$$\begin{array}{c} \begin{array}{c} \text{Score}({\text{B}}^{\prime },\text{Y})=\text{logP}(\text{Y}|{\text{B}}^{\prime })-\alpha \cdot |\text{Y}|-\gamma \cdot \Sigma_{\text{𝓁}\in U}\text{c}({\text{b}}_{\text{𝓁}}^{\prime }) \end{array} \end{array}$$ (25)

Where $logP(Y|B^{\prime })$ represents the conditional probability of text Y given candidate encoding sequence $B^{\prime }$, capturing semantic contextual consistency; α·|Y| is a length regularization term suppressing unreasonably short or long text outputs; $\gamma \cdot \Sigma_{\text{𝓁}\in U}\text{c}({\text{b}}_{\text{𝓁}}^{\prime })$ is a local cost penalty term controlling the aggressiveness of structural repairs; and γ is the weight coefficient.

The final optimal braille-cell sequence $\hat{B}$ and Chinese text $\hat{Y}$ are obtained by maximizing the joint score across candidate sets:


$$\begin{array}{c} \begin{array}{c} \left(\hat{\text{B}},\hat{\text{Y}})=\text{argma}{\text{x}}_{{\text{B}}^{\prime }\in \text{C}(\text{B},U)}\text{ma}{\text{x}}_{\text{Y}}\text{Score}({\text{B}}^{\prime },\text{Y}\right) \end{array} \end{array}$$ (26)

This design keeps disambiguation local, interpretable, and computationally controllable. It also limits the propagation of structural uncertainty by allocating additional contextual reasoning only to positions that remain unreliable after grid-based recovery and structural correction.

### 4.8. Training objectives and optimization

Training follows the same decomposition as the inference pipeline and is organized into two supervised components: structural parsing and sequence generation. Instead of collapsing all decisions into a single objective, optimization is divided into point-level structural supervision and sequence-level text supervision. This design first stabilizes six-dot structural recovery and then strengthens sequence learning at positions where structural uncertainty remains. It is also consistent with the formulation of braille transcription as a structured decoding problem rather than a purely end-to-end mapping.

**(1) Structured parsing module: point-level binary cross-entropy**

Point-level predictions in the grid analysis module are supervised by binary cross-entropy (BCE), which measures the discrepancy between the predicted point-existence probability and the ground-truth dot label:


$$\begin{array}{c} \begin{array}{c} L_{dot}=-\Sigma_{\text{𝓁},\text{i}}\left[{\text{d}}_{\text{𝓁},i}\text{log}{\text{p}}_{\text{𝓁},i}+(\text{1}-{\text{d}}_{\text{𝓁},i})\text{log}(\text{1}-{\text{p}}_{\text{𝓁},i})\right] \end{array} \end{array}$$ (27)

Where ${\text{d}}_{\text{𝓁},i}$ denotes the ground-truth label of the i-th dot position in the 𝓁-th cell, and ${\text{p}}_{\text{𝓁},i}$ denotes the predicted probability that the corresponding location contains a valid braille dot. This loss directly supervises structural recovery at the point level and encourages stable estimation of dot presence under real imaging degradation.

**(2) Sequence generation module: negative log-likelihood and weighted sequence loss**

The text output of the sequence generation module is supervised by negative log-likelihood (NLL), defined over the reference text sequence:


$$\begin{array}{c} \begin{array}{c} L_{\text{seq}}=-\Sigma_{\text{t}}\text{log}\text{P}({\text{y}}_{\text{t}}|{\text{y}}_{<\text{t}},\text{B}) \end{array} \end{array}$$ (28)

Where $y_{t}$ denotes the reference token at decoding step t, $y_{<t}$ denotes the preceding target context, and B denotes the recovered braille representation used for sequence prediction. This loss measures the quality of sequence generation conditioned on the recovered six-dot structure.

To strengthen learning at difficult positions, an additional weighting mechanism is introduced for decoding steps associated with low-confidence braille cells. The weighted sequence loss is written as


$$\begin{array}{c} \begin{array}{c} L_{\text{seq}}^{\text{w}}=-\Sigma_{\text{t}}\omega_{\text{t}}*\text{ logP}({\text{y}}_{\text{t}}|{\text{y}}_{<\text{t}},\text{B}) \end{array} \end{array}$$ (29)

The weighting coefficient is defined as


$$\begin{array}{c} \begin{array}{c} \omega_{\text{t}}=\text{1}+\kappa \cdot I[\text{t is mapped from }U] \end{array} \end{array}$$ (30)

Where U denotes the set of low-confidence braille cells, I[·] is the indicator function, which equals 1 when decoding step t is associated with a braille cell in U and 0 otherwise, and κ is the weight gain coefficient. Under this design, decoding steps associated with structurally uncertain cells receive larger training weights, so that the model pays more attention to positions that are more likely to cause downstream transcription errors.

**(3) Optimization and validation protocol**

Model selection is carried out under document-level five-fold cross-validation. The validation split is used to determine the thresholds for τ and η, the temperature parameter T and the candidate number n. Early stopping is applied to reduce overfitting. Optimization is performed useing AdamW together with cosine annealing of the learning rate to improve convergence stability.

## 5. Experiments

### 5.1. Dataset and annotations

The experimental dataset consists of a synthetic text-to-text parallel corpus derived from the zho_news_2007-2009_1M-sentences.txt dataset, originally sourced from the Leipzig Corpora Collection. Instead of physical documents, the dataset comprises up to 1,000,000 Chinese news sentences that were programmatically translated into Braille Unicode characters using the braille.org.cn translation API. To account for real-world variations in Braille conventions, the dataset includes augmented versions where Braille tone marks are selectively removed at rates of 0%, 90%, and 100%. The final processed sentence pairs are securely aligned and distributed into an 8:1:1 split, yielding 80% for the training set, 10% for the validation set, and 10% for the testing set.

The dataset contains both regular and irregular layout samples. Regular samples follow standard row-column organization, whereas irregular samples include real document disturbances such as paragraph indentation, line breaks, partial occlusion, and minor creases. This design allows the evaluation to reflect not only standard braille layouts but also the structural variability commonly encountered in real documents.

Data organization is defined at the document or page level. During the split into training, validation, and test sets, grouping is performed strictly by document ID, so that pages from the same document are not distributed across different subsets. This prevents data leakage caused by shared paper texture, acquisition conditions, or document-specific artifacts, and ensures that the reported results better reflect cross-document generalization ability.

The annotation protocol includes three levels of supervision, with multiple rounds of review used to reduce annotation noise. The first level consists of candidate braille region boxes for front-end region localization. The second level consists of cell-level six-dot sequences used for structural training and for dot-level and cell-level evaluation. The third level consists of Chinese reference text used for sequence generation and end-to-end evaluation. For samples that contain only Chinese reference text, six-dot sequence annotations are obtained through an initial grid parsing step followed by manual correction of low-confidence positions, which reduces the cost of structural annotation while preserving supervision quality. To ensure high annotation quality, inter-annotator reliability was calculated, achieving a Cohen’s Kappa score of 0.55, indicating moderate agreement between independent annotators.

### 5.2. Validation protocol and metric implementation

Document-level five-fold cross-validation was used to evaluate model performance. All documents were divided into five mutually exclusive subsets. In each fold, one subset was used for testing, one for validation, and the remaining three for training. The validation set was used to select model hyperparameters, including the thresholds τ and η, the temperature parameter T, and the candidate number n, as well as for early stopping. The test set was used only for final result reporting, in order to avoid bias introduced by hyperparameter tuning on test data.

Experimental results are reported as mean ± standard deviation over the five test folds. The calculation formula is:


$$\begin{array}{c} \begin{array}{c} \text{Result}={\text{mean}}_{\text{k}=\text{1}..\text{K}}\left({\text{Metric}}_{\text{k}}\right)\pm {\text{std}}_{\text{k}=\text{1}..\text{K}}\left({\text{Metric}}_{\text{k}}\right) \end{array} \end{array}$$ (31)

To reduce discrepancies caused by implementation details, several consistency controls were applied throughout evaluation. First, Chinese text was normalized using a unified procedure, including full-width and half-width character conversion, whitespace normalization, punctuation cleaning, and synonym mapping where applicable. Second, Dot-F1 was computed under a fixed indexing protocol. For sequence-level evaluation, BLEU-4 tokenization was performed strictly at the Chinese character level to ensure consistency. To assess performance significance among different methods, paired t-tests were utilized with a significance level set at p<0.05. Furthermore, representative error examples—highlighting failure cases in severe ghosting and ambiguous contexts—are provided in the Supplementary Material to further improve interpretability. Third, all compared methods were evaluated under the same data partitioning scheme, metric definitions, and computational budget. This unified evaluation protocol helps ensure that performance differences reflect model behavior rather than inconsistencies in preprocessing or metric implementation.

### 5.3. Baselines

To evaluate the effectiveness of the proposed method, four baseline settings were constructed to represent the main technical alternatives in braille recognition. All baselines were evaluated under the same data partitioning scheme, training protocol, and metric definitions as the proposed method, so that performance differences could be attributed to differences in modeling strategy rather than to inconsistencies in experimental configuration.

**(1) B1: Rule-based decoding + direct text mapping**

This baseline performs braille dot decoding using rule-based structural analysis and directly maps the resulting cell sequence to text, without explicit sequence-level disambiguation. It represents traditional rule-based braille recognition pipelines.

**(2) B2: Dot parsing without structural consistency constraints + sequence generation**

This baseline performs dot-level prediction and sequence generation, but does not apply energy-based structural repair or topological consistency constraints during cell recovery. It represents learning-based methods that rely on local predictions but do not explicitly incorporate braille structural priors.

**(3) B3: Structured dot parsing + sequence generation without sequence-level disambiguation**

This baseline retains structural recovery and sequence generation, but removes candidate expansion and disambiguation for low-confidence positions. It is used to assess the contribution of the uncertainty-guided sequence-level disambiguation module after structural recovery has already been introduced.

**(4) B4: Dot parsing + sequence generation without structural consistency and sequence-level disambiguation**

This baseline removes both the structural consistency module and the uncertainty-guided sequence-level disambiguation module. It relies entirely on the initial independent point-level predictions and base sequence generation. It is used to assess the system’s lower-bound performance when neither topological constraints for visual structural repair nor local context-aware candidate expansion is applied to mitigate imaging degradations.

### 5.4. Main results

Table 1 summarizes the main comparison results of the proposed method and the four baseline settings under document-level five-fold cross-validation. Performance is evaluated jointly using Dot-F1, CER, and BLEU-4 to reflect structural recovery quality, character-level transcription accuracy, and sentence-level consistency, respectively.

**Table 1:** Main results under five-fold cross-validation (mean ± SD across the five test folds).

Method	Dot-F1 (↑)	CER (↓)	BLEU-4 (↑)
B1: Rule-based decoding + direct mapping	0.975 ± 0.006	0.061 ± 0.009	52.3 ± 2.8
B2: No structural consistency constraints	0.984 ± 0.004	0.033 ± 0.005	64.7 ± 1.9
B3: No sequence-level disambiguation	0.990 ± 0.003	0.024 ± 0.004	69.8 ± 1.6
B4: Without structural consistency and sequence-level disambiguation	0.978 ± 0.006	0.067 ± 0.010	49.6 ± 3.1
Proposed method	0.992 ± 0.002	0.018 ± 0.003	74.1 ± 1.2

The proposed method achieves the best performance on all three metrics, with a Dot-F1 of 0.992 ± 0.002, a CER of 0.018 ± 0.003, and a BLEU-4 score of 74.1 ± 1.2. These results indicate that the combination of structured dot parsing, structural consistency repair, and uncertainty-guided sequence disambiguation improves performance not only at the structural level but also in final text transcription. The high Dot-F1 suggests stable recovery of six-dot patterns under real document conditions, while the low CER and high BLEU-4 indicate improved character accuracy and stronger contextual consistency in the generated text.

Among the baseline settings, B1 shows the weakest performance across all three metrics, indicating that rule-based decoding with direct text mapping is insufficiently robust under realistic imaging degradation. B2 improves substantially over B1, but still remains clearly below the proposed method. This result suggests that local dot prediction alone is not sufficient, and that explicit structural consistency constraints play an important role in suppressing the cross-layer propagation of point-level errors. B3 performs closer to the proposed method, which indicates that once structural recovery becomes relatively stable, sequence-level disambiguation remains necessary for resolving residual ambiguity at low-confidence positions. The further improvement from B3 to the full method therefore supports the contribution of the uncertainty-guided local disambiguation module.

In addition, all methods show relatively small standard deviations across folds, while the proposed method yields the smallest variation overall. This suggests that the framework maintains stable performance under document-level partitioning and exhibits stronger cross-document generalization than the baseline settings.

### 5.5. Ablation study

To evaluate the contribution of each core component, ablation experiments were conducted under the same experimental configuration as the full model by removing the structural consistency module and the sequence-level disambiguation module, respectively. The results are summarized in Table 2.

**Table 2:** Ablation results under five-fold cross-validation (mean ± SD across the five test folds).

Experiment Settings	Dot-F1 (↑)	CER (↓)	BLEU-4 (↑)
Full Method	0.992 ± 0.002	0.018 ± 0.003	74.1 ± 1.2
Without sequence-level disambiguation	0.990 ± 0.003	0.024 ± 0.004	69.8 ± 1.6
Without structural consistency	0.984 ± 0.004	0.033 ± 0.005	64.7 ± 1.9
Without both modules	0.978 ± 0.006	0.067 ± 0.010	49.6 ± 3.1

Table 2 shows that the full method achieves the best performance across all three metrics, with a Dot-F1 of 0.992 ± 0.002, a CER of 0.018 ± 0.003, and a BLEU-4 score of 74.1 ± 1.2. Removing either module leads to consistent degradation, indicating that both structural recovery and sequence-level disambiguation contribute to the final transcription quality.

When the sequence-level disambiguation module is removed, Dot-F1 decreases only slightly, from 0.992 to 0.990, whereas CER increases from 0.018 to 0.024 and BLEU-4 decreases from 74.1 to 69.8. This pattern suggests that the main role of sequence-level disambiguation is not to substantially alter structural recovery, but to resolve residual ambiguity after structure has been recovered, thereby improving character-level accuracy and contextual consistency in the generated text.

When the structural consistency module is removed, the degradation is more evident at both the structural and transcription levels. Dot-F1 drops from 0.992 to 0.984, while CER rises to 0.033 and BLEU-4 decreases to 64.7. These results indicate that structural consistency repair is essential for stabilizing six-dot recovery and for suppressing the propagation of point-level errors into later decoding stages. Without this module, the system becomes more sensitive to imaging noise and local structural disturbances.

The largest performance drop appears when both modules are removed simultaneously. In this case, Dot-F1, CER, and BLEU-4 deteriorate to 0.978, 0.067, and 49.6, respectively. This result shows that the two modules are complementary rather than redundant: structural consistency improves the reliability of the recovered braille representation, while sequence-level disambiguation absorbs the residual uncertainty that remains after structural recovery. Their combination is therefore necessary for achieving the full performance of the proposed framework.

### 5.6. Robustness under different imaging conditions

To evaluate robustness under different types of imaging degradation, the test set was partitioned into six groups according to illumination, tilt, and blur conditions. For each group, CER and BLEU-4 were reported together with three diagnostic quantities derived from uncertainty localization: the disambiguation trigger rate r, the low-confidence coverage Cover, and the error concentration rate Err@U. The results are summarized in Table 3.

**Table 3:** Robustness under different imaging conditions.

Imaging condition	CER (↓)	BLEU-4 (↑)	Trigger Rater	Coverage (Cover)	Error Concentration (Err@U)
Uniform illumination	0.016	75.2	5.2%	0.23	0.72
Uneven illumination	0.021	72.8	8.9%	0.28	0.76
Mild tilt	0.017	74.6	6.0%	0.24	0.74
Severe tilt	0.026	69.9	13.7%	0.35	0.81
Mild blur	0.019	73.8	7.8%	0.26	0.77
Severe blur	0.031	66.4	18.5%	0.41	0.85

Table 3 shows that the proposed method performs best under relatively mild conditions, such as uniform illumination and mild tilt, where CER reaches 0.016 and BLEU-4 reaches 75.2. Performance degrades under more challenging conditions, especially severe tilt and severe blur, where CER increases to 0.026 and 0.031, and BLEU-4 decreases to 69.9 and 66.4, respectively. Even so, the method maintains usable transcription performance across all groups, indicating stable robustness under realistic imaging variation.

As image degradation becomes more severe, the disambiguation trigger rate increases from 5.2% under uniform illumination to 18.5% under severe blur. At the same time, Cover rises from 0.23 to 0.41, and Err@U rises from 0.72 to 0.85. This trend suggests that the uncertainty estimates remain responsive to image quality degradation and that a larger proportion of actual transcription errors is concentrated in the low-confidence set under more difficult conditions. In other words, the uncertainty localization mechanism becomes more active precisely when the input becomes less reliable.

The largest degradation is observed under severe tilt and severe blur. This is consistent with two main sources of error: first, distorted or shifted point-response peaks increase grid fitting residuals and reduce structural stability; second, higher point uncertainty produces more ambiguous six-dot interpretations, which places greater pressure on sequence-level disambiguation. The grouped results therefore support the role of the proposed closed-loop design: structural recovery absorbs part of the visual degradation, while local sequence disambiguation focuses additional computation on high-risk positions to reduce the propagation of residual errors.

### 5.7. Parameter sensitivity and inference efficiency

The main adjustable parameters of the proposed method are the disambiguation trigger threshold η, which is set equal to the dot decision threshold τ in the default configuration, and the candidate number n per low-confidence cell. These parameters directly affect the disambiguation trigger rate, transcription accuracy, and additional computational overhead. In addition, end-to-end inference time is decomposed by processing stage in order to identify the main computational bottlenecks and assess the practical cost of local sequence disambiguation. The corresponding results are reported in Table 4 and Table 5.

**Table 4:** Parameter sensitivity under different settings of η and 𝐧.

Parameter Settings (η/n)	Trigger rate	CER (↓)	Additional Overhead (↓)
0.35/3	6.5%	0.020	8%
0.45/3	10.8%	0.018	14%
0.55/3	16.4%	0.017	22%
0.45/5	10.8%	0.017	20%

**Table 5:** End-to-end inference time breakdown (Runtime is reported as average ms/ROI).

Inference Stage	Average runtime	Percentage
ROI Normalization + Point Response Calculation	4.8	13%
Grid Parameterization and Robust Fitting	7.2	19%
Point Sampling + Structural Consistency Repair	10.9	29%
Sequence Generation (without Disambiguation)	12.3	33%
Sequence Disambiguation (Additional Overhead)	2.0	6%

**(1) Parameter sensitivity analysis**

Table 4 reports the disambiguation trigger rate, CER, and additional computational overhead under different combinations of η and n. The results show a clear trade-off between transcription accuracy and inference cost. When the trigger threshold is smaller, the disambiguation module is activated less frequently, which reduces computational overhead but leads to a higher CER. As η increases, both the trigger rate and the additional overhead rise, while CER decreases gradually and then approaches saturation. This trend indicates that the benefit of more aggressive disambiguation becomes limited beyond a certain point.

A similar pattern is observed for the candidate number n. Increasing n from 3 to 5 reduces CER only slightly, from 0.018 to 0.017, whereas the additional computational overhead increases from 14% to 20%. This suggests diminishing marginal gains from enlarging the candidate set. Considering both transcription accuracy and inference cost, the default configuration is set to η=0.45 and n=3. Under this setting, the trigger rate is 10.8%, CER is 0.018, and the additional overhead is 14%, providing a balanced trade-off between accuracy improvement and computational efficiency.

**(2) End-to-end inference efficiency breakdown**

Table 5 reports the average runtime and relative proportion of each stage in end-to-end inference, measured in ms/ROI. The results show that the main computational bottlenecks are sequence generation without disambiguation and point sampling with structural consistency repair, which account for 33% and 29% of the total runtime, respectively. In contrast, the additional overhead introduced by sequence-level disambiguation is only 2.0 ms/ROI, or 6% of the total processing time.

These results suggest that the proposed local disambiguation strategy improves transcription quality without introducing substantial extra cost. Because candidate expansion is restricted to low-confidence positions, the method avoids the combinatorial growth associated with full-sequence expansion while maintaining controllable inference overhead. ROI normalization and grid fitting occupy relatively small portions of the total runtime, which further supports the practicality of the overall framework for deployment-oriented braille document transcription.

## 6. Conclusions

This study formulates Chinese braille document transcription as a structured decoding problem rather than a purely end-to-end image-to-text task. A closed-loop inference framework is introduced to combine structural recovery, uncertainty estimation, and sequence-level disambiguation. By separating visual structural recovery from semantic generation, the method reconstructs valid six-dot patterns under topological constraints, produces dot-level and cell-level confidence estimates for risk localization, and applies local candidate expansion only at low-confidence positions. This design reduces the propagation of point-level errors while keeping the additional inference cost under control.

A document-level evaluation protocol is used to validate the proposed framework through five-fold cross-validation, baseline comparison, ablation analysis, robustness analysis under different imaging conditions, parameter sensitivity analysis, and inference efficiency breakdown. The proposed method achieves a Dot-F1 of 0.992 ± 0.002, a CER of 0.018 ± 0.003, and a BLEU-4 score of 74.1 ± 1.2, while maintaining stable performance across folds and under varying document conditions. In addition, the extra overhead introduced by sequence-level disambiguation is limited to 6% of the total inference time, supporting the practical applicability of the framework to deployment-oriented braille document transcription. The intermediate diagnostic outputs, including low-confidence positions and candidate scores, also improve interpretability and may assist manual proofreading and engineering risk control.

Several directions remain for future work. First, the cross-domain adaptation mechanism can be strengthened through improved calibration of thresholds and temperature parameters under more challenging photographic conditions. Second, richer structural priors and lightweight learned constraints may further improve robustness to severe deformation, blur, and ghosting. Third, the uncertainty-guided local disambiguation strategy can be extended to more complex linguistic and layout phenomena, including punctuation, numerals, special symbols, and cross-line structures, in order to support more accurate transcription of long and structurally complex braille documents.
